# Sonographic presentation of rice bodies in subacromial-subdeltoid chronic bursitis

**DOI:** 10.1186/s13089-019-0130-y

**Published:** 2019-07-22

**Authors:** Raham Bacha, Iqra Manzoor, Syed Amir Gilani

**Affiliations:** 1 University Institute of Radiological Sciences and Medical Imaging Technologies (UIRSMIT), Faculty of Allied Health Sciences (FAHS), The University of Lahore, Lahore, Pakistan; 2 University Institute of Radiological Sciences and Medical Imaging Technologies (UIRSMIT), The University of Lahore, G 10\2 Canal Berg, Lahore, 54000 Pakistan; 3 Dean Faculty of Allied Health Sciences (FAHS), The University of Lahore, Lahore, Pakistan

**Keywords:** Rice bodies, Shoulder joint, Bursitis, Ultrasound, subacromial-subdeltoid bursa

## Abstract

**Background:**

subacromial-subdeltoid chronic bursitis is one of the main causes of shoulder pain syndrome. There are various causes of shoulder pain syndrome including chronic bursitis. The sonographic appearances of chronic bursitis vary from patient to patient, depending upon the underlying cause. However, rice bodies are the rare sonographic presentation among them. Rice bodies can be associated with atypical mycobacterial tenosynovitis, bursitis, mycobacterium tuberculosis and infections, but very rarely occur in the chronic subacromial-subdeltoid bursitis. Its unique sonographic presentation makes it easy to be diagnosed confidently, but it is difficult to be recognized, interpret and distinguish from other pathologies due to its rare occurrence. It is therefore intended to present this rare and interesting sonographic presentation of chronic subacromial-subdeltoid bursitis as a case report.

**Case-presentation:**

A 36-year-old male suffering from swelling and pain on his left shoulder for 6 months came to the clinic with Grade-3 impingement syndrome. Free-floating echogenic rice bodies were identified in the subacromial-subdeltoid bursa during ultrasound examination, which was also confirmed with Magnetic resonance imaging (MRI) and serological tests of the specimen after surgery.

**Conclusion:**

The unique sonographic presentation of rice bodies in the chronic subacromial-subdeltoid bursitis makes it easy to be diagnosed confidently, but it is difficult to be recognized, due to its rare occurrence.

**Electronic supplementary material:**

The online version of this article (10.1186/s13089-019-0130-y) contains supplementary material, which is available to authorized users.

## Introduction

Shoulder pain is a disabling clinical entity contributing to a great extent to the individual dependency. It is the third most common musculoskeletal complaint in orthopedic practice [1]. There are various causes of shoulder pain syndrome ranging from capsulitis to tendon tear and joint dislocation. Chronic bursitis is one of the main causes of shoulder pain; however, the sonographic presentation of the shoulder pain varies from individual to individual due to underlying causes [2]. Rice bodies are a rare and unique sonographic presentation of chronic bursitis. In chronic bursitis, whitish rice resembling biconcave grains are formed; hence, named as rice bodies [3]. It can also be formed during chronic rheumatoid arthritis and also seen in patients without any underlying causes [4]. They can be formed in different joints of the body including shoulder, knee, wrist, elbow and ankle, but the joint which is affected mostly is shoulder and knee joint. Rheumatoid arthritis is a common complication during which rice bodies can be formed and they are also present in patients without any underlying causes [5]. Under the microscope, the rice bodies are seen as white and shiny grains of rice while on histological examination they are seen as inner amorphous core of acidophilic material which is surrounded by fibrin and collagen. It is often debated, that rice bodies are a nonspecific response to synovial inflammation [6]. The exact pathophysiology of the rice bodies is still unknown. According to some researchers, the rice bodies arise from the microinfarcts of the synovial membrane which leads to the shedding of the synovium [7]. Contrary to this, some researchers say that these bodies are produced impulsively in the synovial fluid and enlarge progressively with the accumulation of the fibrin [8]. Rice bodies are the products of the acute and chronic inflammation of the synovium, but they are not the part of chondroid tissues. Infection can also be ruled out when the count of WBC is in normal physiological range [9]. Rice bodies can be detected with the help of different imaging modalities including plain X-ray, Computed tomography (CT) scan, MRI and Ultrasound. Sonoelastography is also useful in the detection of various musculoskeletal pathologies with sensitivity and specificity of 100% and 89%, respectively [10]. However, the advent of state-of-the-art modality and technological development in ultrasound makes it more helpful in the evaluation of superficial structures and their pathologies [11]. As diagnostic ultrasound has no bioeffects and is free of radiation hazards, it is justified to conduct more and more studies on the application of ultrasound in the diagnosis of various diseases.

## Case-presentation

**Fig. 1:**
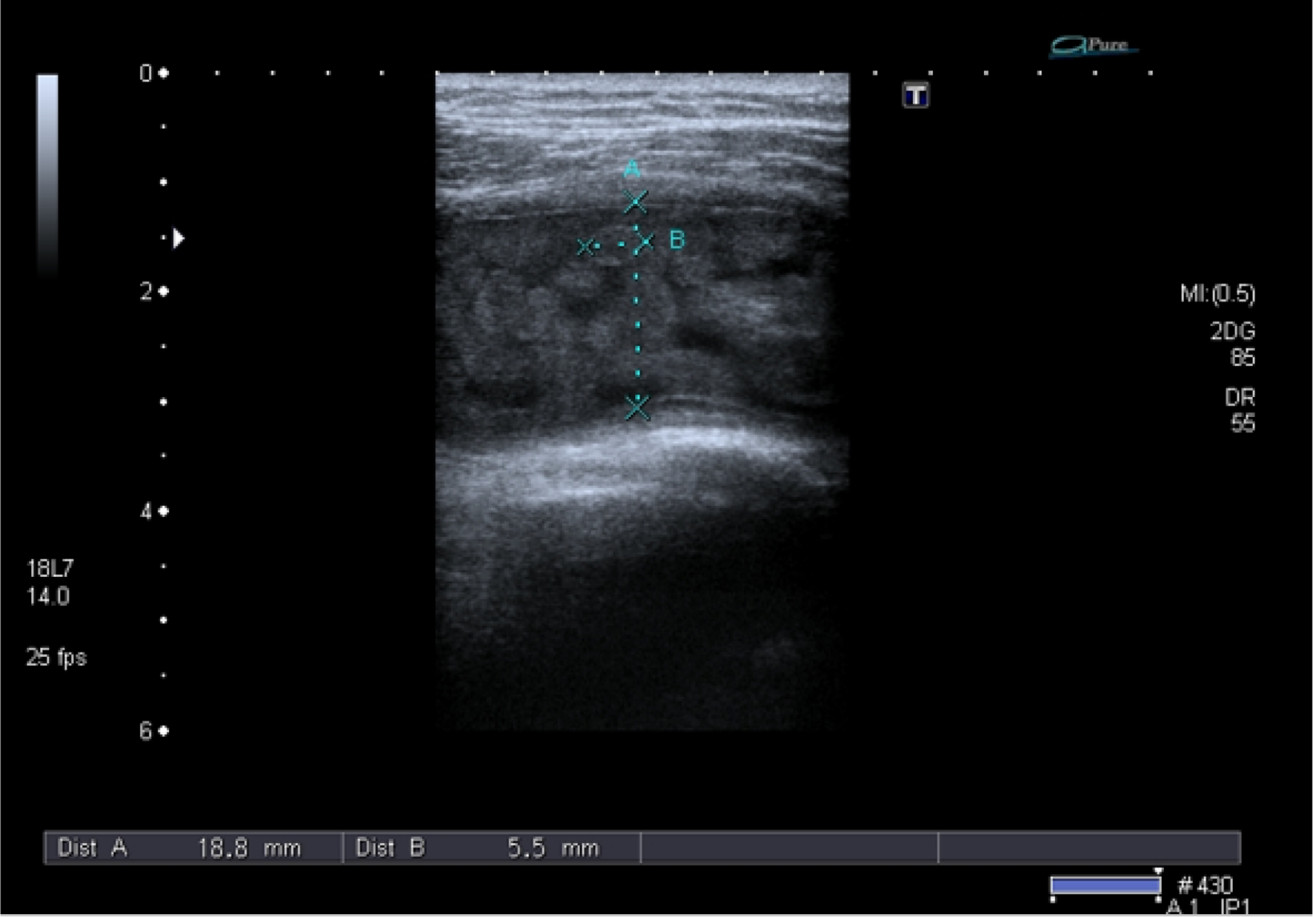
Rice bodies in the subacromial-subdeltoid bursa

**Fig. 2:**
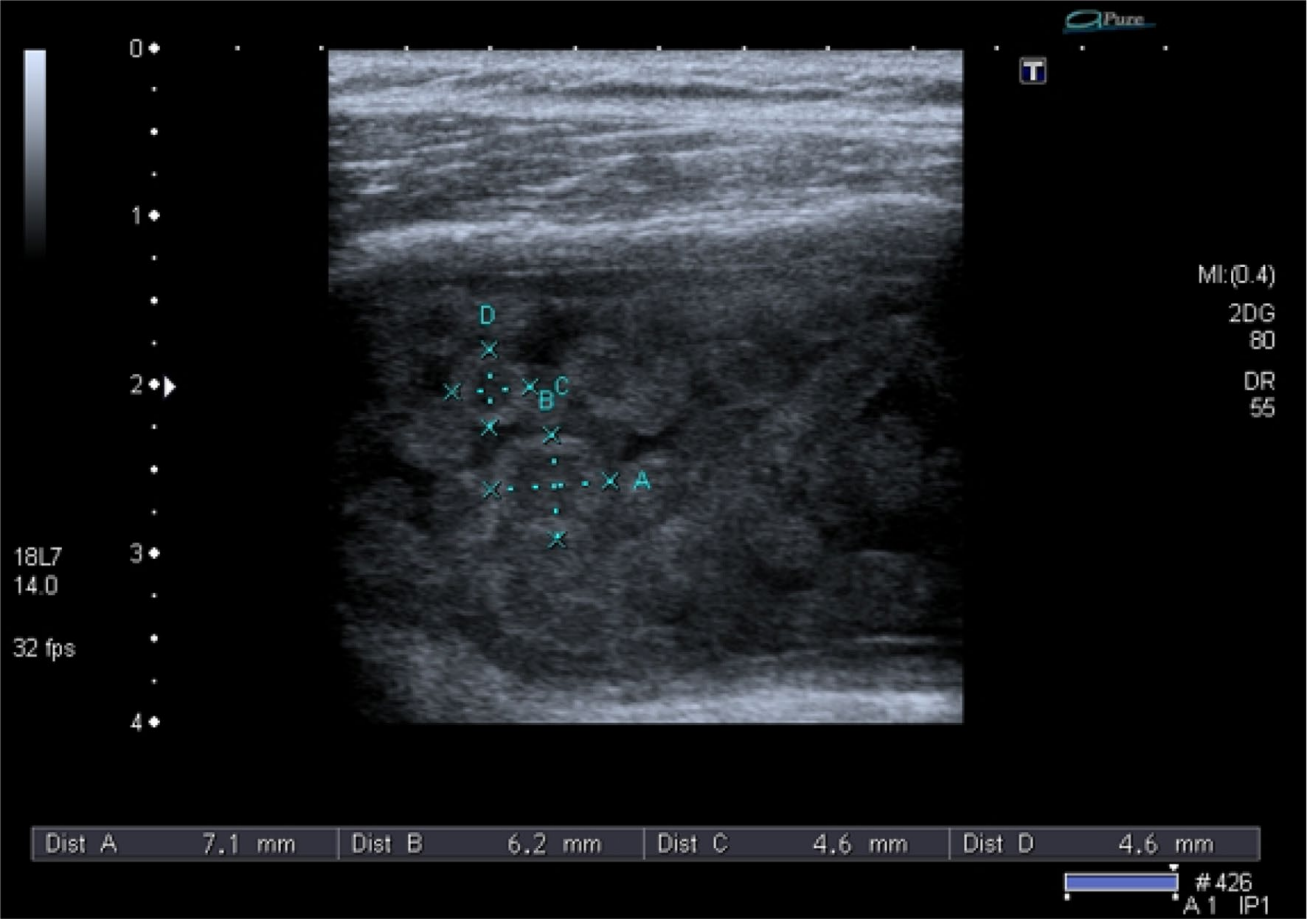
Measurement of the rice bodies

**Fig. 3:**
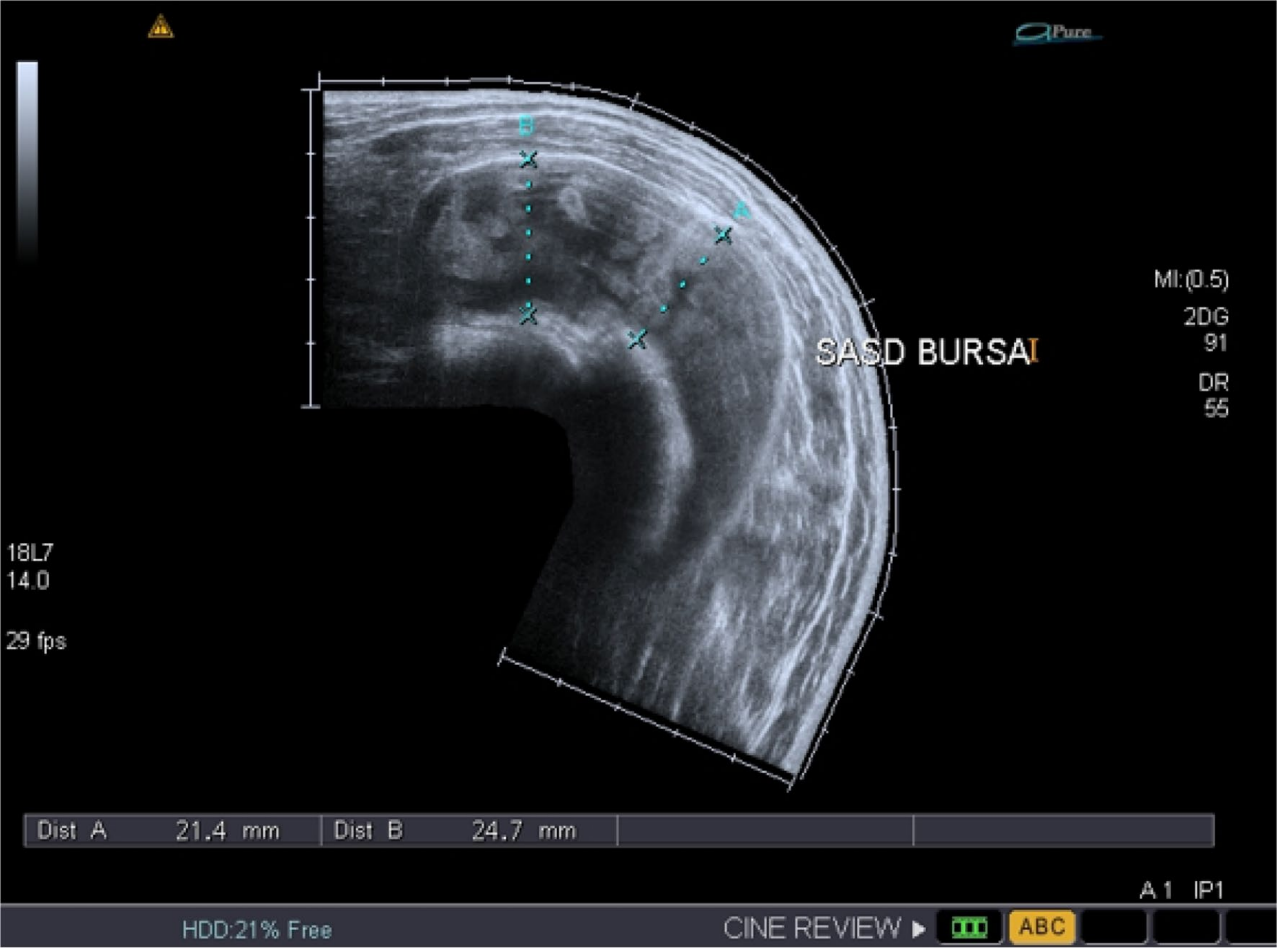
Panoramic view of the rice bodies

A 36-year-old married male came to the Gilani ultrasound center, Lahore, Pakistan. He was a medical lab assistant by profession. On physical examination, he was suffering from shoulder pain for last 6 months but his pain was aggravated from last 1 month and his pain was so severe that he was not able to lie on the affected shoulder. No fever, warmth, and change in color were noted on the affected shoulder. Prominent difference between two shoulders and compressible swelling on the affected shoulder was seen during the scan. On laboratory examination, erythrocyte sedimentation rate (ESR) of the patient was not significantly raised (21 mm/h). The white blood cells (WBC’s) were in normal range, Rheumatoid factor (RF) was negative, and C-reactive protein (CRP) concentration level and urine analysis were normal. The patient was scanned in sitting position with the real-time ultrasound examination technique with Toshiba (Xario 200) with linear transducer frequency ranging from 7 to 14 MHz. Left bursa was massively enlarged while measuring from 18 to 24 mm in vertical dimension (Figs. 1, 3). There were multiple biconcave bodies like RBCs with moderate echogenicity in the subacromial-subdeltoid bursa (Fig. 1). These bodies were freely moving and compressible with the transducer compression and joint movement (Additional file 1: Video S1). The size of the rice bodies was measured as 4.6 to 7.1 mm (Fig. 2). Mid-range movement was impaired but movement became normal while abducted with the help of contralateral hand. The cracking sound was also heard with active shoulder movement. The moment of the glenohumeral joint was restricted having grade-3 impingement. Panoramic view of the subacromial-subdeltoid bursa showing numerous round lentil-like bodies which are named as rice bodies was also taken (Fig. 3). No tear was found in the rotator cuff tendon. Posterior glenoid labrum was normal. Long head of biceps tendon was found in the bicipital groove and was normal. Deltoid muscle and pectoralis muscles were normal (Additional file 1: Video S1). X-ray was also performed after the ultrasound examination which revealed no abnormalities except soft tissue swelling. Next day, MRI was performed which confirms the presence of rice bodies in the subacromial-subdeltoid bursa.

## Discussion

The rice bodies were first described by Riese in association with tuberculosis arthritis about a century ago [12]. They are termed as rice bodies because of their close resemblance with the shape and size of the polished rice grains. A great variation is seen in the prevalence of the rice bodies in patients of rheumatoid arthritis, but in one study reported that they are found microscopically as high as 72% [13]. Significant improvement in the complaints of the patients was evident with surgical removal of rice bodies from the affected bursa [3]. The differential diagnosis made for these tiny echogenic materials revealed pigmented villonodular synovitis and synovial chondromatosis especially in the latter stages. In primary synovial chondromatosis, normal joints can be affected but the rice bodies can be associated with tuberculous arthritis, rheumatoid arthritis, osteoarthritis and infective arthritis. This is because synovial chondromatosis results due to metaplasia of subsynovial connective tissue into cartilage nodules. If the rice bodies are mineralized, they can easily be visualized on plain radiographs. Otherwise, MR T-2-weighted scans are very helpful for its diagnosis [14]. An article published in 1996 by Griffith et al. presented 2 cases of the subacromial-subdeltoid bursitis with rice bodies in the size of the rice bodies ranging from 3 to 10 mm, while in our study, the size was ranging from 4.6 to 7.1 mm [15]. Another study reported a similar case of subacromial bursitis with multiple small rice bodies. The findings of X-ray, MRI and Ultrasound were compared for the diagnosis of rice bodies [16]. Similarly, a case was reported by Adriana and colleagues in 2012, while observing a massive subacromial-subdeltoid bursitis with rice bodies secondary to an orthopedic implant. A large encapsulated fluid was found in the subacromial-subdeltoid bursa, which contained several hundred small 5-mm rice bodies [17]. Various protocols and indications are applicable in different countries of the world. With the prudent use of ultrasound, various diseases could be diagnosed easily which were previously not included in the spectra of its diagnosis [18].Our case report demonstrated a rare case of isolated subacromial-subdeltoid bursitis with rice bodies; the size and appearance were similar to the international studies. But the name rice bodies are suitable for these particles in the subacromial-subdeltoid bursa due to its white color but the sonographic appearance of these bodies was more round-like lentils (Figs. 1, 2, 3).

## Conclusion

The unique sonographic presentation of rice bodies in the chronic subacromial-subdeltoid bursitis makes it easy to be diagnosed confidently, but it is difficult to be recognized, due to its rare occurrence.

## Additional file

**Additional file 1: Video S1.** Additional videos.

## Data Availability

The data of the patient were obtained from our private clinic.

## Abbreviations

MRI: magnetic resonance imaging
CT: computed tomography
WBCs: white blood cells
ESR: erythrocyte sedimentation rate
RF: rheumatoid factor
CRP: c-reactive protein
SASD bursa: subacromial-subdeltoid bursa
